# The Anti-Proliferative Effects of Enterolactone in Prostate Cancer Cells: Evidence for the Role of DNA Licencing Genes, mi-R106b Cluster Expression, and PTEN Dosage

**DOI:** 10.3390/nu6114839

**Published:** 2014-11-03

**Authors:** Mark J. McCann, Ian R. Rowland, Nicole C. Roy

**Affiliations:** 1 Food Nutrition & Health, Food and Bio-based Products, AgResearch Grasslands Research Centre, Palmerston North 4442, New Zealand; E-Mail: nicole.roy@agresearch.co.nz; 2 Gravida: National Centre for Growth and Development, The University of Auckland, Auckland 1142, New Zealand; 3 Department of Food and Nutritional Sciences, P.O. Box 226, Whiteknights, Reading RG6 6AP, UK; E-Mail: i.rowland@reading.ac.uk; 4 The Riddet Institute, Massey University, Palmerston North 4442, New Zealand

**Keywords:** enterolactone, lignan, prostate, proliferation, PTEN, miR-106b cluster

## Abstract

The mammalian lignan, enterolactone, has been shown to reduce the proliferation of the earlier stages of prostate cancer at physiological concentrations *in vitro*. However, efficacy in the later stages of the disease occurs at concentrations difficult to achieve through dietary modification. We have therefore investigated what concentration(s) of enterolactone can restrict proliferation in multiple stages of prostate cancer using an *in vitro* model system of prostate disease. We determined that enterolactone at 20 μM significantly restricted the proliferation of mid and late stage models of prostate disease. These effects were strongly associated with changes in the expression of the DNA licencing genes (GMNN, CDT1, MCM2 and 7), in reduced expression of the miR-106b cluster (miR-106b, miR-93, and miR-25), and in increased expression of the PTEN tumour suppressor gene. We have shown anti-proliferative effects of enterolactone in earlier stages of prostate disease than previously reported and that these effects are mediated, in part, by microRNA-mediated regulation.

## 1. Introduction

Prostate cancer is the second most common cancer in men worldwide with seventy percent of annual diagnoses occurring in Westernised societies [1]. The incidence of the disease is considerably higher in the EU, North America and New Zealand than in China (14, 22 and 25-fold, respectively) [2,3,4,5] Whilst risk factors for prostate cancer such as age, ethnic origin and heredity are important, geographical and economic differences in diet and lifestyle appear to influence prostate disease risk to a greater extent [6,7,8]. A clear link between diet and prostate cancer is yet to be shown, due in part to a lack of understanding of the effects, or absence of effect of dietary components on the mechanisms of prostate tumourigenesis.

Enterolactone (ENL) is a weakly-oestrogenic (100 to 1000-fold less compared to natural oestradiol) mammalian metabolite that is produced by the metabolism of plant lignans by intestinal bacteria, but may also be present in low amounts in dairy foods and meat as a consequence of ruminant intestinal metabolism (reviewed [9,10,11]), [12,13,14,15,16]. ENL has been reported to have anti-cancer, anti-oxidant, anti-inflammatory and anti-angiogenic properties [10,11,17,18,19,20,21], but ecological studies examining ENL exposure and disease risk, especially with regard to prostate cancer, have been inconclusive [9,10,11]. This is due, in part, to a lack of understanding of how the inter-individual response to ENL may be affected by diet and lifestyle, genetic and/or epigenetic factors and intestinal microbiota composition.

Serum or urinary ENL levels, a biomarker of exposure, vary considerably by population and dietary preference, and typically ranges from 0.1 to 10 μM [9,10,11]. There is, however, some evidence that ENL can accumulate to higher levels (up to 25-fold higher) in prostate tissue and fluid, suggesting a biological function for ENL in the prostate [22]. Although there are human, animal and *in vitro* studies showing that purified ENL, or foods rich in ENL, can inhibit the development and progression of prostate cancer for example by reducing proliferation [18,19,20,21] or affecting steroid metabolism and activity [23], it is not yet clear if these effects occur at concentrations achievable through dietary intake alone [9,10,11]. There is a distinct lack of data available on the concentration of ENL in prostate tissue pre and post-intervention with ENL precursors, which restricts our understanding of how bio-available ENL is in the prostate. We have recently shown that physiologically-relevant concentrations of ENL can reduce the proliferation of early-stage prostate disease *in vitro* and that these effects are associated with alterations in the expression of DNA replication licencing genes [19].

The correct initiation of DNA replication requires the licencing of origin of replications by the minichromosome maintenance complex (MCM) [24]. The loading of this MCM complex is facilitated, in part, by chromatin licensing and DNA replication factor 1 (CDT1), which is itself negatively regulated by geminin (GMMN). Abnormal expression of GMNN, CDT1, and MCM2 and 7 have been linked with the malignant progression of prostate cancer [25,26,27,28,29,30,31]. Another key signalling pathway disrupted in prostate cancer is the phosphoinositide-3-kinase (PI3K)-AKT signalling pathway. The phosphatase and tensin homolog (PTEN) tumour suppressor gene negatively regulates the PI3K/AKT pathway and PTEN is one of the most common tumour suppressor genes whose appropriate function is compromised in prostate cancer (~70% of cases) [32,33,34], which leads to abnormal proliferation and cell death. Initiation of DNA replication and PTEN tumour suppression are transcriptionally-linked as one of the MCMs (MCM7) has a microRNA cluster (miR-106b, -93, and 25) in one of its introns that suppresses PTEN translation and dysregulation of the cluster is also linked to cancer [35].

Cancer is not composed of abnormal cells at the same stage of disease; rather it is a series of abnormal cells at differing stages of disease that collectively compromise the appropriate function of the tissue. Previous research has shown anti-proliferative effects of ENL in one or in limited stages of disease, rather than efficacy in a range of disease states [18,19,20,21]. Therefore, we hypothesised that ENL could restrict the proliferation of more than just the later stages of prostate disease. To investigate our hypothesis we used an *in vitro* model system of six prostate cell lines representing the early (RWPE-1 and WPE1-NA22), mid (WPE1-NB14 and WPE1-NB11) and later (WPE1-NB26 and LNCaP) stages of prostate tumourigenesis [36,37]. The LNCaP cell line is a model of the switch between androgen sensitivity and insensitivity during prostate disease that occurs in the later stages of carcinogenesis [37]. This *in vitro* model system was used to assess how the metabolic activity, growth rate, cell cycle progression changes with ENL exposure over 24 and 48 h. Based on these data we explored potential mechanisms for the anti-proliferative activity by measuring the expression of the GMMN, CDT1, MCM2 and MCM7, miR-106b cluster, and PTEN genes.

## 2. Experimental Section

### 2.1. Cell Culture and Enterolactone Preparation

Authenticated RWPE-1 (P52), WPE1-NA22 (P20), WPE1-NB14 (P16), WPE1-NB11 (P24), WPE1-NB26 (P15), and LNCaP (P22) cell lines were purchased from the American Type Culture Collection (Manassas, VA, USA) to test the effects of ENL. All cell culture reagents were obtained from Life Technologies (Auckland, New Zealand) unless otherwise stated. The cell lines were cultured and maintained as described previously [19], with all experiments were completed within ten sub-cultures from the original ATCC stock.

A stock solution of ENL (45199, Sigma-Aldrich, Auckland, New Zealand) at 16.76 mM (100% DMSO) was prepared and used to prepare test concentrations of ENL in cell-line specific medium. Etoposide (Sigma-Aldrich, Auckland New Zealand) was used as a positive control for proliferation as it blocks DNA synthesis resulting in apoptosis. The negative control for all experiments was cell-line specific medium adjusted to contain 0.36% v/v of DMSO.

### 2.2. Cell Viability—Mitochondrial Activity Assay

The effect of 10 to 100 μM ENL on the metabolic activity of the six cell lines over 48 h was measured using the water soluble tetrazolium cytotoxicity assay (WST-1, Clontech, Mountain View, CA, USA) as described previously [19]. The negative control and each concentration of ENL were tested on twenty-four biological replicates for both time points. The absorbance of the formazan dye produced was measured at 450 and 650 nm using a SpectraMax 250 spectrophotometer (Molecular Devices, Sunnyvale, CA, USA). For all measurements (including the blank), the background absorbance (650 nm) was subtracted from the detection wavelength (450 nm) and these corrected values were used for analysis.

### 2.3. Cell Viability—Growth Kinetics Assay

The effect of 10 to 60 μM ENL on the viability of each cell line over 48 h was measured using trypan blue staining and the number of non-blue (viable) cells counted as described previously [19]. For each of three separate assays for each time point, each concentration was tested on two technical replicates with an initial seeding density of 5 × 10^5^ cells. From these data the effect of ENL on the doubling time of the cell lines was calculated.

### 2.4. Cell Viability—Cell Cycle Profile Assay

The effect of 20 μM ENL on the cell cycle profile of six cell lines over 48 h was measured with the Dead Cell Apoptosis Kit with Annexin V Alexa Fluor^®^ 488 & Propidium Iodide from Life Technologies (Auckland, New Zealand). This kit is a flow cytometry kit used to measure early apoptosis by detecting phosphatidyl serine expression and membrane permeability [18,38].

Each assay was completed according to the manufacturer’s instructions. For each of three separate assays for each time point, each concentration was tested on three technical replicates with an initial seeding density of 5 × 10^5^ cells. The fluorescence intensities of Alexa Fluor 488 and Propidium Iodide in each sample, at 585 nm, were measured using a FACSCalibur flow cytometer with CELLQuest Pro Software (BD Biosciences, Auckland, New Zealand), and analysed using FlowJo V7.6.3 (TreeStar, Ashland, OR, USA).

### 2.5. Quantification of Gene Expression—mRNA and miRNA Genes

The expression of GMNN, CDT1, MCMs 2 and 7, PTEN, hsa-miR-106b, hsa-miR-93, and hsa-miR-25 by the six cell lines treated with 20 μM ENL over 48 h was quantified using probe-based real-time PCR. All reagents were obtained from Life Technologies (Auckland, New Zealand) unless otherwise stated. For each gene and time point, two biological replicates (with triplicate qPCR measurements) of 5 × 10^5^ cells were used.

The NucleoSpin^®^ miRNA kit (Macherey-Nagel, Düren, Germany) was used to extract large and small RNA in separate fractions from each of the samples according to the manufacturer’s instructions. RNA quantity and integrity was determined based on A260:280 and A260:230 nm ratios using a NanoDrop 1000 spectrophotometer (Thermo Fisher Scientific, Melbourne, Australia) and a Agilent 2100 bioanalyser (Agilent, Santa Clara, CA, USA). Only RNA with both absorbance ratios of 1.8 to 2.1 and with a RIN value of 9 or greater were considered to be of sufficient quality and integrity.

For the large RNA fractions, 500 ng was reverse transcribed into cDNA using a high capacity RNA-to-cDNA kit according to the manufacturer’s instructions. The expression levels of the mRNA transcripts of the GMNN, CDT1, MCMs 2 and 7, and PTEN genes were quantified using pre-validated PrimeTime Nuclease assays (Hs.PT.51.14706721.g, Hs.PT.53.27448129.gs, Hs.PT.53.25820936, Hs.PT.53.23112694.g, and Hs.PT.51.14706721.g) (Integrated DNA Technologies, Singapore, Singapore). The HPRT1 (Hs.PT.39a.22214821) reference gene was used to normalise for RNA content.

For the small RNA fractions, 10 ng was reverse transcribed into cDNA with gene specific primers using the TaqMan microRNA RT kit according to the manufacturer’s instructions. The expression levels of the mature hsa-miR-106, hsa-miR-93, and hsa-miR-25 genes were quantified using pre-validated TaqMan assays (000442, 002139, and 002442). The RNU6B reference gene (001093) was used to normalise for RNA content.

All real-time PCR assays were prepared as triplicate 10 μL reactions comprising a 9.0 μL aliquot of master mix (5.0 μL of 2x Kapa Fast Probe mix (Kapa Biosystems, Wilmington, DE, USA), 0.5 μL of 20x mRNA or miRNA gene assay, 3.5 μL of nuclease-free water, and 1 μL of cDNA (10-fold dilution in nuclease-free water). The thermal profile used was: 95 °C for 20 s, followed by 40 cycles of 95 °C for 3 s and 60 °C for 30 s. The experiment was completed using a RotorGene 6000 qPCR instrument (Qiagen, Hilden, Germany). Data were normalised to the appropriate reference gene and analysed for expression level changes (ratio compared to untreated) using the ΔCq method with efficiency correction. The efficiencies for all PCRs ranged between 1.91 and 2.03, where 2.0 represents 100% efficiency.

### 2.6. Statistical Analyses

All data were analysed for statistical significance using a one-way ANOVA with SigmaStat 12.3 (Systat Software Inc., San Jose, CA, USA). The normality of the data was tested using the Shapiro-Wilk method and the equality of variance using the Leven Median test. Non-normally distributed data was ranked and analysed using the Kruskal-Wallis ANOVA method. Following ANOVA, significantly different means were identified using the Dunnett’s post-hoc test. A probability (*p*) value of less than 0.05 was considered to show a significant difference.

## 3. Results

### 3.1. ENL Reduces the Viability of Mid to Later Stage Prostate Disease Cell Lines

**Figure 1:**
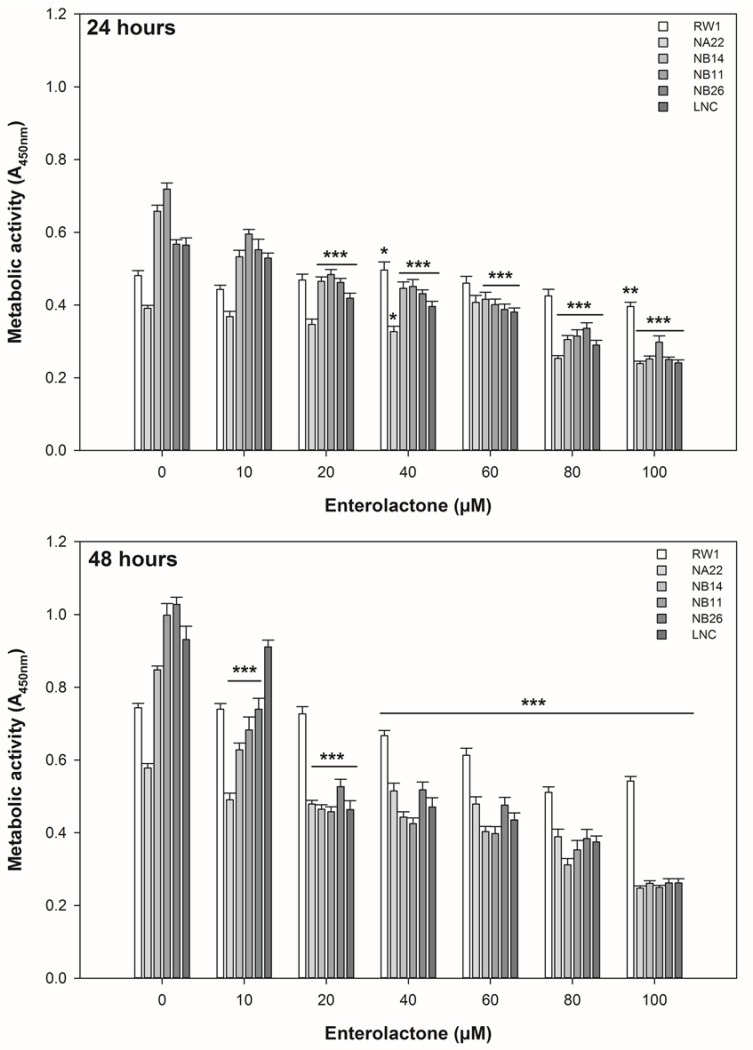
The effect of ENL on the metabolic activity of prostate cell lines over 48 h. The data are expressed as the mean absorbance ±SEM (*n* = 24). A statistical difference between untreated and treated samples is indicated by * (*p* < 0.05), ** (*p* < 0.01), or *** (*p* < 0.002).

**Figure 2:**
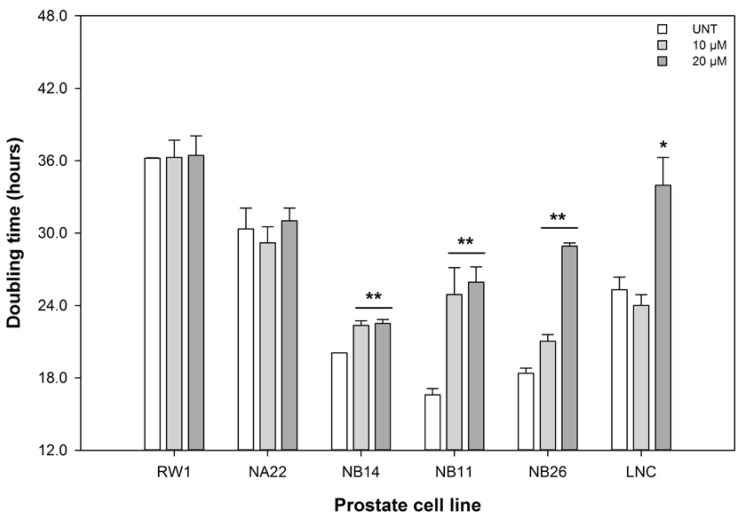
The effect of ENL on the doubling times of prostate cell lines over 48 h. The data are expressed as the mean doubling time ±SEM (*n* = 3). A statistical difference between untreated (UNT) and treated samples is indicated by * (*p* < 0.05), or ** (*p* < 0.01).

ENL exerted differential effects on the mitochondrial metabolic activity and growth kinetics of the prostate cell lines at 24 and 48 h of exposure (Figure 1 and Figure 2).

At 24 h, 20 μM ENL or greater significantly reduced the metabolic activity of the WPE1-NB14, WPE1-NB11, WPE1-NB26, and LNCaP cell lines. At 48 h, 40 μM ENL or greater reduced the activity of all cell lines. However, the activity of the WPE1-NA22 (10 and 20 μM), WPE1-NB14 (10 and 20 μM), WPE1-NB11 (10 and 20 μM), WPE1-NB26 (10 and 20 μM), and LNCaP (20 μM) cell lines was reduced at this time point. The RWPE-1 cell line tolerated up 40 μM ENL without significant alterations in metabolic activity. The metabolic activity of the WPE1-NB44, WPE1-NB11, WPE1-NB26, and LNCaP cell lines were significantly reduced in a dose dependent manner at concentrations of 20 μM of greater at both time points. The WPE1-NB14 and WPE1-NB11 cells were particularly sensitive to ENL at 24 and 48 h.

These data indicate that the lowest concentration of ENL that affects the metabolic activity in the “diseased” cell lines, but does not affect the “normal” cell line is 20 μM. As 40 to 100 μM ENL clearly affected all cell lines, these concentrations were excluded from further analysis. As changes in metabolic activity may result in altered growth rates, *i.e*., a change in the time taken for a population of cells to double in number, we measured the doubling times of the cell lines in response to 10 and 20 μM ENL over 48 h.

The growth kinetics, based on the time for the population to double in number, of the RWPE-1 and WPE1-NA22 cell lines were unaltered by ENL. The positive control, 20 μM etoposide, significantly (*p* < 0.018) decreased the metabolic activity and increased in the doubling time of the cell lines over 48 h. The WPE1-NB14, WPE1-NB11, and WPE1-NB26 cell lines were the most sensitive to the ENL-induced increased doubling time (*i.e*., slower growth) of these cell lines. The doubling time of the LNCaP cell line was only affected by 20 μM ENL.

As 20 μM ENL was the lowest concentration that affected both the metabolic activity and doubling times of the WPE1 and LNCaP cell lines, but not the RWPE-1 cell line (the least diseased cell line in our model and an approximation of a “normal” cell line) this concentration was selected for further study.

### 3.2. ENL Restricts the Cell Cycle of and Induces Apoptosis in Mid to Later Stage Prostate Disease Cell Lines

**Figure 3:**
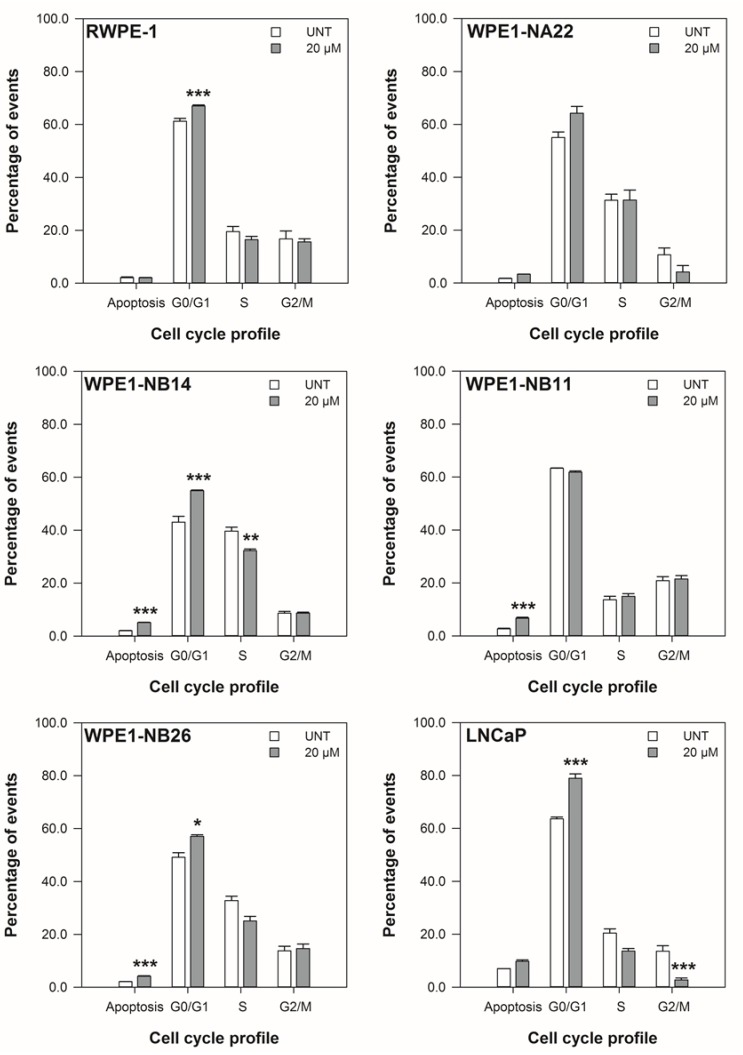
The effect of 20 μM ENL on the cell cycle profile of prostate cell lines after 24 h. The data are expressed as the mean percentage of events in each phase ±SEM (*n* = 3). A statistical difference between untreated and treated samples is indicated by * (*p* < 0.05), ** (*p* < 0.01), or *** (*p* < 0.002).

**Figure 4:**
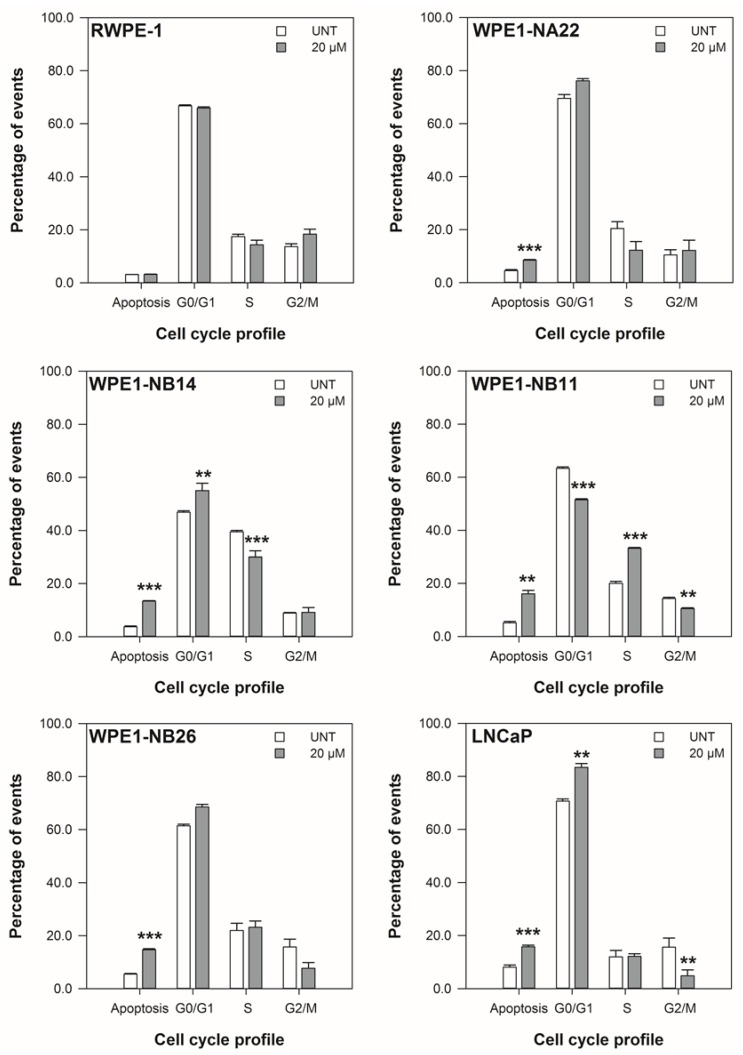
The effect of 20 μM ENL on the cell cycle profile of prostate cell lines after 48 h. The data are expressed as the mean percentage of events in each phase ±SEM (*n* = 3). A statistical difference between untreated and treated samples is indicated by * (*p* < 0.05), ** (*p* < 0.01), or *** (*p* < 0.002).

The restriction of cell cycle progression in the cell lines with 20 μM ENL over 48 h is shown in Figure 3 to Figure 4. The positive control, 20 μM etoposide, significantly increased the S-phase and level of apoptosis of the cell lines over 48 h (*p* < 0.014).

At 24 h, there was an increase in the percentage of cells in the G_0_/G_1_ phase of the cell cycle for the RWPE-1, WPE1-NB14, WPE1-NB11, WPE1-NB26, and LNCaP cell lines in response to 20 μM ENL. For the LNCaP cell line there was also decrease in the percentage of cells in the G_2_/M phase. At 48 h, the cell cycle of the WPE1-NB14 and WPE1-NB11 cell lines remained altered (NB14: increased G_0_/G_1_, decreased S, and NB11: decreased G_0_/G_1_, increased S, decreased G_2_/M) by ENL. The G_0_/G_1_ and G_2_/M phases of the LNCaP cell line were also restricted, both reduced, after 48 h.

The data in Figure 3 and Figure 4 also show that 20 μM ENL induces apoptosis in the WPE1-NB14, WPE1-NB11, and WPE1-NB26 after 24 and 48 h. At 48 h, the WPE1-NA22 and LNCaP cell lines also had increased levels of apoptosis in response to ENL.

These data indicate that the disrupted viability (metabolic activity and doubling times), shown in Figure 1 and Figure 2, of the cell lines is due, in part, to alterations in cell cycling and cell death. Given the alterations shown Figure 3 and Figure 4, the effect of 20 μM ENL on the expression of genes involved in two key pathways during abnormal growth and carcinogenesis was quantified to explore potential mechanisms of action.

### 3.3. ENL Alters the Expression of DNA Licencing Genes in Mid to Later Stage Prostate Disease Cell Lines

**Figure 5:**
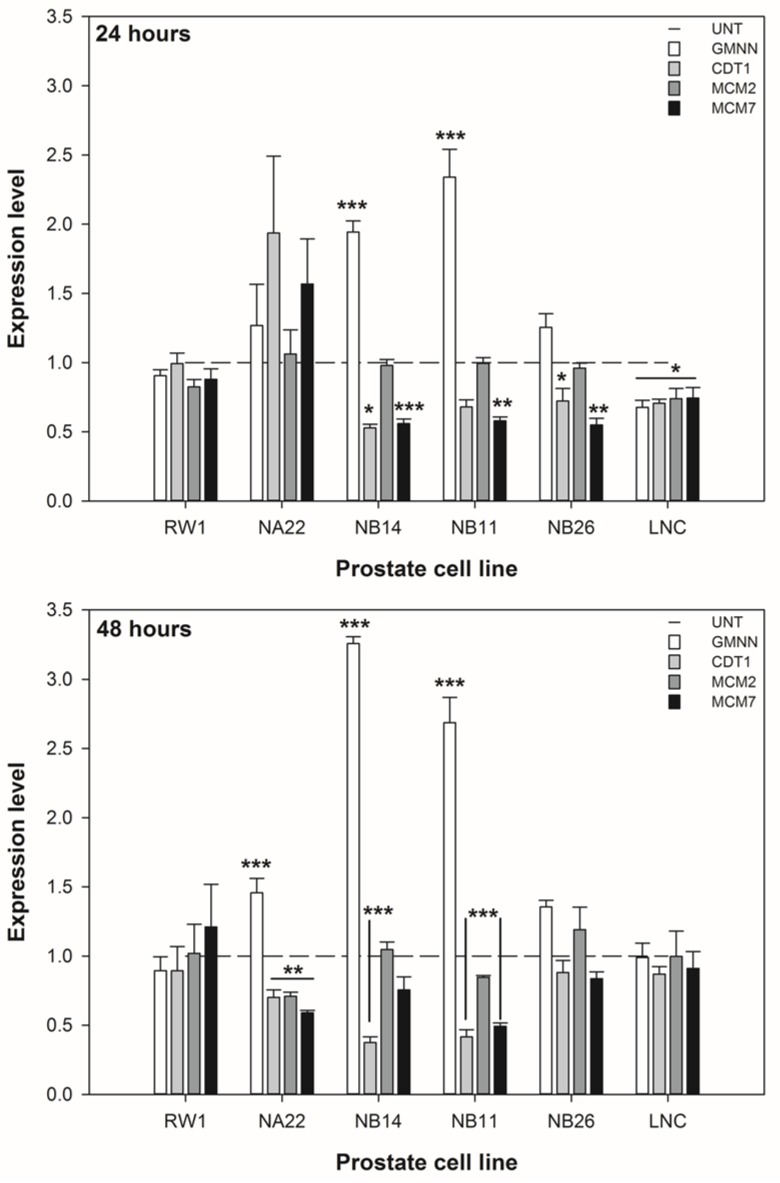
The effect of 20 μM ENL on the expression of DNA licencing genes by prostate cell lines over 48 h. The data are expressed as the mean expression level ±SEM (*n* = 3). For each cell line, a difference between untreated and treated samples is indicated by * (*p* < 0.05), ** (*p* < 0.01), or *** (*p* < 0.002).

The expression of the DNA licencing genes in response to 20 μM ENL by the six cell lines are shown in Figure 5. The co-efficient of variation for the HPRT1 reference gene amongst the untreated cell lines was 9% and 5%, at 24 and 48 h respectively. These data show that the expression of GMNN (CDT1 inhibitor) is increased approximately 2 to 3 fold in the WPE1-NB14 and WPE1-NB11 cell lines after 24 and 48 h. The expression of CDT1 was reduced in these cell lines by approximately 2 fold. The expression of the MCM2 and 7 genes was reduced in the majority of cell lines at 24 h, but only in the WPE1-NA2, WPE1-NB14, and WPE1-NB11 cell lines at 48 h. These changes in expression imply that the licencing of DNA for replication is reduced and would results in cell cycle restrictions (particularly in the G_0_/G_1_ and S phases), reduced proliferation, and/or increased cell death.

The reduced expression of MCM7 suggests that the miR-106b cluster (located in one of the introns of MCM7) may also be influenced by ENL and if so this may affect the expression of the PTEN gene.

### 3.4. ENL Alters the Expression of the miR-106b Cluster Leading to Increased PTEN Expression

**Figure 6:**
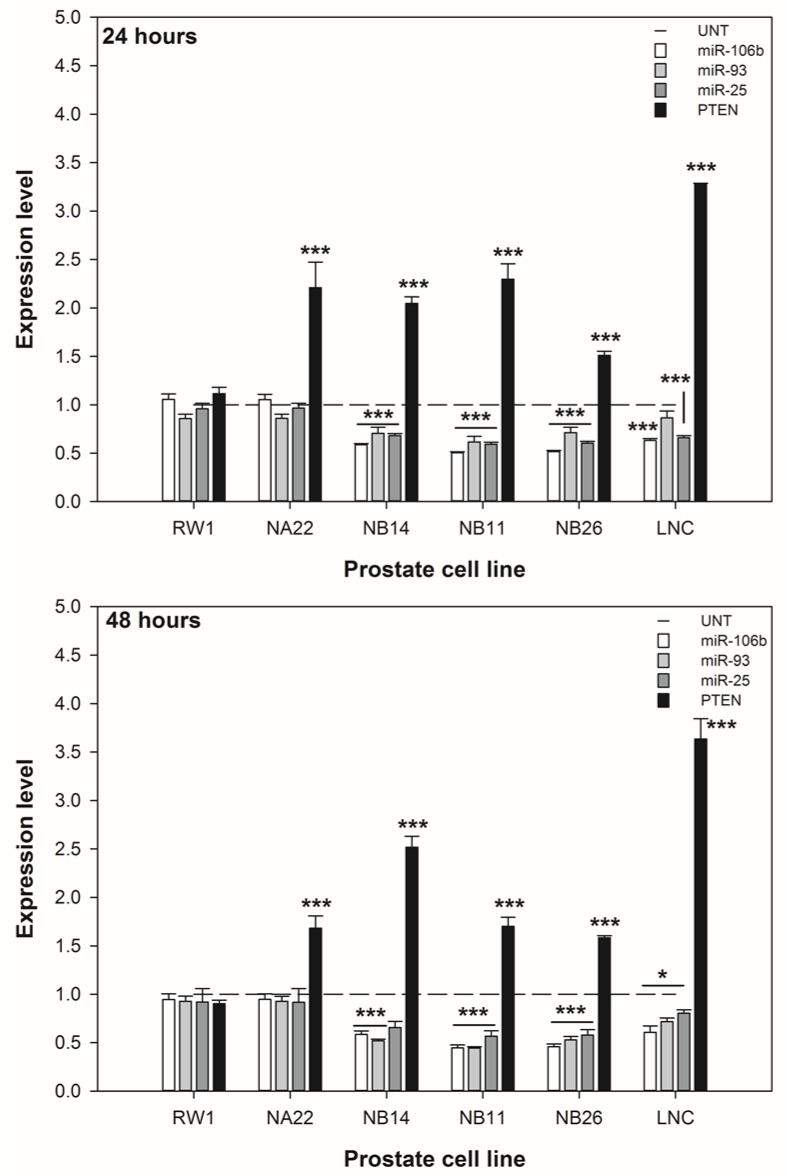
The effect of ENL on the expression of the PTEN gene and miR-106b cluster genes by prostate cell lines over 48 h. The data are expressed as the mean expression level ±SEM (*n* = 3). For each cell line, a difference between untreated and treated samples is indicated by * (*p* < 0.05), or *** (*p* < 0.002).

The expression of the miR-106b cluster and PTEN genes in response to 20 μM ENL by the six cell lines are shown in Figure 6. The co-efficient of variation for the rnu6b reference gene in the untreated cell lines was 2.3% and 2%, at 24 and 48 h respectively. These data show that the expression of miR-106b, miR-93, and miR-25 are decreased in the WPE1-NB14, WPE1-NB11, WPE1-NB26, and LNCaP cell lines after 24 and 48 h. The expression of PTEN is substantially increased in the WPE1 and LNCaP cell lines.

These data suggest that the repression of the miR-106b cluster leads, in part, to increased PTEN expression. However, the expression of PTEN was increased by ENL in the WPE1-NA22 cell line despite no substantial change in the expression of the miR-106b cluster.

## 4. Discussion

The present study provides further evidence that a pure mammalian lignan inhibits the *in vitro* proliferation of prostate cell lines. To our knowledge, this is the first study to examine the correlation between the expression of genes associated with DNA licencing and miR-106b cluster mediated PTEN and biological end-points of proliferation *in vitro*.

Previous *in vitro* studies examining how the prostate cell lines respond to ENL have reported anti-proliferative effects for concentrations ranging from 25 to 100 μM and our data are in agreement with these findings [18,20]. In contrast to the previous studies we have studied the effects of ENL on the proliferation of a range of prostate cell models, rather than the late-stage models generally used, and explored potential mechanisms of action. We have shown that ENL at 20 μM over 48 h is sufficient to restrict the proliferation of primarily mid to later stage prostate cancer cells without any effects on the approximately “normal” RWPE-1 cell line. However, this cell line is immortalised and is not truly normal. Additionally we have shown that the anti-proliferative effects of ENL are strongly associated with: (1) improved negative regulation of abnormal DNA licencing (increased GMNN expression and decreased CDT1 expression); and (2) inhibition of miR-106b cluster expression leading to increased expression of the tumour suppressive gene PTEN.

The ENL-induced changes in genes required for DNA replication initiation may explain the effects of ENL on cell cycle control and consequently proliferation in the prostate cell lines. However, the effect of altering the GMNN/CDT1 balance in tumourigenic cells (which express higher levels of these genes compared to normal cells [31]) is unclear as there is debate about how the GMNN/CDT1 balance influences the development and progression of cancer [26,27,28,39,40]. Additionally we have also shown that the expression of MCM2 and MCM7 is reduced by ENL in the prostate cell lines used, and this may be linked altered CDT1 expression as CDT1 is required for the loading of the MCM complex during the initiation of DNA replication [24]. MCM7, in particular, is known to be oncogenic [25,29,41] not only for its role in DNA licencing, but also due to other interactions such as: (1) MCM7 overexpression can inhibit the retinoblastoma-controlled cell G_1_/S cell cycle block [42,43]; (2) MCM7 interacts with the androgen receptor [44], appropriate androgen signalling and the consequences of androgen insensitivity are key factors in prostate carcinogenesis and relapse [45,46]; and (3) one of the introns of MCM7 contains the mir-106b cluster [35] which targets two key tumour suppressor genes implicated in the prostate cancer, PTEN and CDKN1A (p21) [47,48,49].

Reduced PTEN expression can contribute to cancer development due to decreased negative regulation of the PI3K/AKT pathway (known as quasi-sufficiency) [50]. Unlike the classic “two-hit” model of tumour suppression, it is the amount of functional PTEN (which can be affected by several factors not just transcription) that determines its tumour suppressive capacity. We have shown that ENL can increase PTEN expression, which may restore the appropriate regulation by PTEN of the PI3K/AKT pathway. However, the LNCaP cell line has a mutated and non-functional PTEN gene [32] and therefore the consequence of its increased expression by ENL is unclear.

The concentrations used in this study have yet to be shown to be achievable in the prostate *in vivo* either through dietary or pharmacological intervention. We also have only demonstrated a link between gene expression and proliferation markers—further work is needed to establish whether the expression changes result in functional changes at the post-transcriptional level. We have shown that there appears to be a relationship between ENL-mediated expression of the PTEN gene, perhaps via suppression of the miR-106b cluster, and proliferation in prostate cancer cell lines. If these are confirmed at the proteomic and functional level, it may represent a novel mechanism for the anti-proliferative activity of ENL.

## 5. Conclusions

In conclusion we have provided evidence for the anti-proliferative effects of ENL in mid and late prostate cell lines, and have shown that changes in the transcription of DNA licencing, miR-106b cluster, and PTEN genes may be involved in these effects. This is important as we have shown that ENL is effective in earlier stages of prostate cancer than previously reported and two important pathways in prostate tumourigenesis are linked through miRNA effects.

## References

[1] Jemal A., Bray F., Center M.M., Ferlay J., Ward E., Forman D. (2011). Global cancer statistics. CA Cancer J. Clin..

[2] Center M.M., Jemal A., Lortet-Tieulent J., Ward E., Ferlay J., Brawley O., Bray F. (2012). International variation in prostate cancer incidence and mortality rates. Eur. Urol..

[3] Ferlay J., Shin H.R., Bray F., Forman D., Mathers C., Parkin D.M. (2010). Estimates of worldwide burden of cancer in 2008: GLOBOCAN 2008. Int. J. Cancer.

[4] Baade P.D., Youlden D.R., Krnjacki L.J. (2009). International epidemiology of prostate cancer: Geographical distribution and secular trends. Mol. Nutr. Food Res..

[5] Cullen J., Elsamanoudi S., Brassell S.A., Chen Y., Colombo M., Srivastava A., McLeod D.G. (2012). The burden of prostate cancer in Asian nations. J. Carcinog..

[6] Venkateswaran V., Klotz L.H. (2010). Diet and prostate cancer: Mechanisms of action and implications for chemoprevention. Nat. Rev. Urol..

[7] Khan N., Afaq F., Mukhtar H. (2010). Lifestyle as risk factor for cancer: Evidence from human studies. Cancer Lett..

[8] Muller D.C., Severi G., Baglietto L., Krishnan K., English D.R., Hopper J.L., Giles G.G. (2009). Dietary patterns and prostate cancer risk. Cancer Epidemiol. Biomark. Prev..

[9] Saarinen N.M., Tuominen J., Pylkkanen L., Santti R. (2010). Assessment of information to substantiate a health claim on the prevention of prostate cancer by lignans. Nutrients.

[10] Adlercreutz H. (2007). Lignans and human health. Crit. Rev. Clin. Lab. Sci..

[11] McCann M.J., Gill C.I., McGlynn H., Rowland I.R. (2005). Role of mammalian lignans in the prevention and treatment of prostate cancer. Nutr. Cancer.

[12] Kuhnle G.G., Dell’Aquila C., Aspinall S.M., Runswick S.A., Mulligan A.A., Bingham S.A. (2008). Phytoestrogen content of foods of animal origin: Dairy products, eggs, meat, fish, and seafood. J. Agric. Food Chem..

[13] Setchell K.D., Lawson A.M., Mitchell F.L., Adlercreutz H., Kirk D.N., Axelson M. (1980). Lignans in man and in animal species. Nature.

[14] Woting A., Clavel T., Loh G., Blaut M. (2010). Bacterial transformation of dietary lignans in gnotobiotic rats. FEMS Microbiol. Ecol..

[15] Clavel T., Borrmann D., Braune A., Dore J., Blaut M. (2006). Occurrence and activity of human intestinal bacteria involved in the conversion of dietary lignans. Anaerobe.

[16] Clavel T., Henderson G., Engst W., Dore J., Blaut M. (2006). Phylogeny of human intestinal bacteria that activate the dietary lignan secoisolariciresinol diglucoside. FEMS Microbiol. Ecol..

[17] Saarinen N.M., Thompson L.U. (2010). Prolonged administration of secoisolariciresinol diglycoside increases lignan excretion and alters lignan tissue distribution in adult male and female rats. Br. J. Nutr..

[18] McCann M.J., Gill C.I., Linton T., Berrar D., McGlynn H., Rowland I.R. (2008). Enterolactone restricts the proliferation of the LNCaP human prostate cancer cell line *in vitro*. Mol. Nutr. Food Res..

[19] McCann M.J., Rowland I.R., Roy N.C. (2013). Anti-proliferative effects of physiological concentrations of enterolactone in models of prostate tumourigenesis. Mol. Nutr. Food Res..

[20] Chen L.H., Fang J., Li H., Demark-Wahnefried W., Lin X. (2007). Enterolactone induces apoptosis in human prostate carcinoma LNCaP cells via a mitochondrial-mediated, caspase-dependent pathway. Mol. Cancer Ther..

[21] Lin X., Switzer B.R., Demark-Wahnefried W. (2001). Effect of mammalian lignans on the growth of prostate cancer cell lines. Anticancer Res..

[22] Morton M.S., Chan P.S., Cheng C., Blacklock N., Matos-Ferreira A., Abranches-Monteiro L., Correia R., Lloyd S., Griffiths K. (1997). Lignans and isoflavonoids in plasma and prostatic fluid in men: Samples from Portugal, Hong Kong, and the United Kingdom. Prostate.

[23] Adlercreutz H., Bannwart C., Wahala K., Makela T., Brunow G., Hase T., Arosemena P.J., Kellis J.T., Vickery L.E. (1993). Inhibition of human aromatase by mammalian lignans and isoflavonoid phytoestrogens. J. Steroid Biochem. Mol. Biol..

[24] Masai H., Matsumoto S., You Z., Yoshizawa-Sugata N., Oda M. (2010). Eukaryotic chromosome DNA replication: Where, when, and how?. Annu. Rev. Biochem..

[25] Luo J.H. (2011). Oncogenic activity of MCM7 transforming cluster. World J. Clin. Oncol..

[26] Hook S.S., Lin J.J., Dutta A. (2007). Mechanisms to control rereplication and implications for cancer. Curr. Opin. Cell Biol..

[27] Lau E., Tsuji T., Guo L., Lu S.H., Jiang W. (2007). The role of pre-replicative complex (pre-RC) components in oncogenesis. FASEB J..

[28] Montanari M., Macaluso M., Cittadini A., Giordano A. (2006). Role of geminin: from normal control of DNA replication to cancer formation and progression?. Cell Death Differ..

[29] Ren B., Yu G., Tseng G.C., Cieply K., Gavel T., Nelson J., Michalopoulos G., Yu Y.P., Luo J.H. (2006). MCM7 amplification and overexpression are associated with prostate cancer progression. Oncogene.

[30] Honeycutt K.A., Chen Z., Koster M.I., Miers M., Nuchtern J., Hicks J., Roop D.R., Shohet J.M. (2006). Deregulated minichromosomal maintenance protein MCM7 contributes to oncogene driven tumorigenesis. Oncogene.

[31] Xouri G., Lygerou Z., Nishitani H., Pachnis V., Nurse P., Taraviras S. (2004). Cdt1 and geminin are down-regulated upon cell cycle exit and are over-expressed in cancer-derived cell lines. Eur. J. Biochem..

[32] Chen Z., Trotman L.C., Shaffer D., Lin H.K., Dotan Z.A., Niki M., Koutcher J.A., Scher H.I., Ludwig T., Gerald W. (2005). Crucial role of p53-dependent cellular senescence in suppression of Pten-deficient tumorigenesis. Nature.

[33] Pourmand G., Ziaee A.A., Abedi A.R., Mehrsai A., Alavi H.A., Ahmadi A., Saadati H.R. (2007). Role of PTEN gene in progression of prostate cancer. Urol. J..

[34] Vlietstra R.J., van Alewijk D.C., Hermans K.G., van Steenbrugge G.J., Trapman J. (1998). Frequent inactivation of PTEN in prostate cancer cell lines and xenografts. Cancer Res..

[35] Poliseno L., Salmena L., Riccardi L., Fornari A., Song M.S., Hobbs R.M., Sportoletti P., Varmeh S., Egia A., Fedele G. (2010). Identification of the miR-106b~25 microRNA cluster as a proto-oncogenic PTEN-targeting intron that cooperates with its host gene MCM7 in transformation. Sci. Signal..

[36] Webber M.M., Quader S.T., Kleinman H.K., Bello-DeOcampo D., Storto P.D., Bice G., DeMendonca-Calaca W., Williams D.E. (2001). Human cell lines as an *in vitro*/*in vivo* model for prostate carcinogenesis and progression. Prostate.

[37] Horoszewicz J.S., Leong S.S., Chu T.M., Wajsman Z.L., Friedman M., Papsidero L., Kim U., Chai L.S., Kakati S., Arya S.K. (1980). The LNCaP cell line—A new model for studies on human prostatic carcinoma. Prog. Clin. Biol. Res..

[38] Van Engeland M., Nieland L.J., Ramaekers F.C., Schutte B., Reutelingsperger C.P. (1998). Annexin V-affinity assay: A review on an apoptosis detection system based on phosphatidylserine exposure. Cytometry.

[39] Arentson E., Faloon P., Seo J., Moon E., Studts J.M., Fremont D.H., Choi K. (2002). Oncogenic potential of the DNA replication licensing protein CDT1. Oncogene.

[40] Blow J.J., Gillespie P.J. (2008). Replication licensing and cancer—A fatal entanglement?. Nat. Rev. Cancer.

[41] Lei M. (2005). The MCM complex: Its role in DNA replication and implications for cancer therapy. Curr. Cancer Drug Targets.

[42] Sterner J.M., Dew-Knight S., Musahl C., Kornbluth S., Horowitz J.M. (1998). Negative regulation of DNA replication by the retinoblastoma protein is mediated by its association with MCM7. Mol. Cell. Biol..

[43] Mukherjee P., Winter S.L., Alexandrow M.G. (2010). Cell cycle arrest by transforming growth factor beta1 near G1/S is mediated by acute abrogation of prereplication complex activation involving an Rb-MCM interaction. Mol. Cell. Biol..

[44] Shi Y.K., Yu Y.P., Zhu Z.H., Han Y.C., Ren B., Nelson J.B., Luo J.H. (2008). MCM7 interacts with androgen receptor. Am. J. Pathol..

[45] Evans C.P., Lara P.N. (2014). Prostate cancer: Predicting response to androgen receptor signalling inhibition. Nat. Rev. Urol..

[46] Balk S.P. (2014). Androgen receptor functions in prostate cancer development and progression. Asian J. Androl..

[47] Song M.S., Salmena L., Pandolfi P.P. (2012). The functions and regulation of the PTEN tumour suppressor. Nat. Rev. Mol. Cell Biol..

[48] Sarker D., Reid A.H., Yap T.A., de Bono J.S. (2009). Targeting the PI3K/AKT pathway for the treatment of prostate cancer. Clin. Cancer Res..

[49] Abbas T., Dutta A. (2009). p21 in cancer: Intricate networks and multiple activities. Nat. Rev. Cancer.

[50] Berger A.H., Knudson A.G., Pandolfi P.P. (2011). A continuum model for tumour suppression. Nature.

